# PERSONALITY AND WORD RECALL AS INTERACTIVE PREDICTORS OF PERCEIVED MEMORY ABILITY

**DOI:** 10.1093/geroni/igad104.1303

**Published:** 2023-12-21

**Authors:** Patrick Hill, Gabrielle Pfund, Patrick Cruitt, Isaiah Spears, Sara Norton, Ryan Bogdan, Thomas Oltmanns

**Affiliations:** Washington University in St. Louis, St. Louis, Missouri, United States; Northwestern University, Chicago, Illinois, United States; Minneapolis VA Health Care System, Minneapolis, Minnesota, United States; Washington University in St. Louis, St. Louis, Missouri, United States; Washington University in St. Louis, St. Louis, Missouri, United States; Washington University in St. Louis, St. Louis, Missouri, United States; Washington University in St. Louis, St. Louis, Missouri, United States

## Abstract

Cognitive gerontology research requires consideration of both actual performance and perceptions of performance. While subjective memory perceptions are positively associated with memory performance, these correlations typically are modest in magnitude. Therefore, researchers have noted the need to consider potential moderators of this association, to better understand whether certain people show weaker or stronger linkages between performance and perceptions. The current study leveraged personality (NEO Big Five), memory performance (i.e., word recall), and perceptions of memory ability (i.e., metamemory in adulthood scale and subjective memory complaints) data from the St. Louis Personality and Aging Network (SPAN) study (n = 774, mean age: 71.52 years). Personality traits were more associated with subjective than objective memory, and the most consistent associations were for extraversion (r = -.20 for complaints, r = .26 for metamemory) and conscientiousness (r = -.26 for complaints and r = .33 for metamemory). Moreover, extraversion moderated associations between word recall and both memory capacity and complaints. Results suggested that associations between word recall and the two subjective indicators were weaker for those adults higher in extraversion, relative to adults who scored lower on extraversion. One possible interpretation is that extraverted older adults may construct their cognitive self-perceptions using information from their social interactions more than objective cues. These findings highlight the need to determine how personality traits influence which sources of information older adults employ when thinking about their cognitive ability, which in turn may explain their choices for activity engagement.

